# Dissecting the Opposing Roles of Thermal Intensity and Growing Degree Days in Regulating Spring Wheat Protein Content

**DOI:** 10.3390/plants15071096

**Published:** 2026-04-02

**Authors:** Xuan Lei, Jun Ye, Xiaobing Wang, Wenjia Yang, Haibin Zhang, Xuanwei Zhao, Juan Liu, Tingjia Zhang, Zhenyu Zhang, Tingyu Ma, Cundong Li, Xin Gao, Juan Li, Zhanyuan Lu

**Affiliations:** 1 School of Life Sciences, Inner Mongolia University, Ministry of Education, Hohhot 010021, China; 15271197763@163.com (X.L.); yejun66@126.com (J.Y.); 2 Key Laboratory of Black Soil Conservation and Utilization, Ministry of Agriculture and Rural Affairs, Key Laboratory of Ecological Restoration and Pollution Control of Degraded Farmland of Inner Mongolia Autonomous Region, Inner Mongolia Academy of Agricultural & Animal Husbandry Sciences, Hohhot 010031, China; xbwang1120@163.com (X.W.); 15248195630@163.com (H.Z.); zxw9339@163.com (X.Z.); m15548752127@163.com (J.L.); z15248891080@163.com (T.Z.); zhangzhenyuwork@126.com (Z.Z.); mty1220@126.com (T.M.); 3 College of Agronomy, Hebei Agricultural University, Baoding 071001, China; nxylcd@hebau.edu.cn; 4 The Middle Reaches of the Yangtze River (Co-Construction by Ministry and Province), Hubei Key Laboratory of Waterlogging Disaster and Agricultural Use of Wetland, College of Agriculture, Yangtze University, Jingzhou 434025, China; wenjiayang123@126.com; 5 College of Agriculture, Inner Mongolia Minzu University, Tongliao 028000, China; gxrty2009@126.com

**Keywords:** spring wheat, protein content, climatic factors, structural equation modeling

## Abstract

Protein content (PC) stability is crucial for wheat quality. This study utilized partial least squares regression and structural equation modeling to distinguish the physiological effects of “thermal intensity” versus “thermal accumulation” on spring wheat PC across Inner Mongolia. Environmental factors were the dominant drivers of variation. Notably, the Erguna region achieved the highest PC (18.53%) despite recording the lowest total growing degree days. Structural equation modeling analysis revealed that thermal intensity during heading-to-anthesis exerted a strong positive effect on PC (path coefficient = 0.965), likely by enhancing nitrogen remobilization kinetics. Conversely, excessive thermal accumulation and sunshine duration during grain filling negatively impacted PC via a carbohydrate-driven “dilution effect”. These findings suggest that superior PC formation requires a specific spatiotemporal coupling: high thermal intensity prior to anthesis to prime nitrogen transport, combined with low thermal accumulation post-anthesis to restrict carbon dilution. This study provides a physiological basis for optimizing wheat quality zoning by decoupling heat magnitude from duration under future climate scenarios.

## 1. Introduction

Wheat (*Triticum aestivum* L.) accounts for approximately 20% of the total protein intake for the human population, playing a pivotal role in global food security. With the escalating market demand for superior processing quality, enhancing protein content (PC) has become a primary breeding objective [1]. However, PC is a complex quantitative trait characterized by high phenotypic plasticity [2], making it highly susceptible to fluctuations in environmental conditions. This leads to instability in quality across different years and regions. Previous research has established that wheat protein content is governed by genotype (G), environment (E), and their complex interactions (G × E) [3,4]. Evidence from multi-environment trials (METs) consistently demonstrates that environmental factors, particularly climatic variability, account for a substantially larger proportion of PC variance than genetic background alone [5,6]. A central, and unresolved controversy in crop physiology is determining the specific thermal drivers of this variability: does grain protein respond more to the magnitude of temperature events during critical windows (“Thermal Intensity”) or to the total heat load over the growing season (“Thermal Accumulation”)?

Nitrogen (N) availability is a primary determinant of grain protein content, as N is a key constituent of amino acids and proteins [7]. Adequate N supply, particularly during the reproductive stages, significantly enhances the activity of nitrogen-assimilating enzymes such as glutamine synthetase (GS) and glutamate synthase (GOGAT), thereby promoting the translocation of nitrogen from vegetative tissues to the developing grain [8]. However, the efficiency of N utilization is highly dependent on environmental conditions, which can modulate the source–ink relationship and the kinetics of N remobilization. Understanding how climatic factors interact with N metabolism is therefore essential for optimizing nitrogen management strategies to achieve high-quality wheat production.

“Thermal Accumulation,” typically quantified by growing degree days (GDD), represents the duration and total quantity of heat resources [9]. Classic source–sink theory posits that higher heat accumulation accelerates phenological development, but often favors carbohydrate assimilation (starch synthesis) over nitrogen deposition [10]. This disproportionate accumulation of starch leads to a “dilution effect,” potentially reducing the final protein concentration [11]. Conversely, “Thermal Intensity” refers to the strength of temperature (e.g., maximum daily temperature, T_max_) during specific developmental stages. Physiologically, nitrogen remobilization from vegetative organs to grains is an enzymatic process governed by kinetics [12]. Higher thermal intensity—within an optimal physiological range—may enhance the activity of key enzymes such as glutamine synthetase (GS), thereby accelerating nitrogen transport rates independent of the total growth duration [13].

Despite the theoretical distinction between these two thermal pathways, empirical studies often conflate them, relying on average temperatures throughout the entire growing season to predict grain quality. This “black-box” approach masks the ontogenetic heterogeneity of crop responses. For instance, a high cumulative heat load might shorten the grain-filling period (negative for quality), while a high temperature intensity at anthesis might prime nitrogen remobilization (positive for quality) [14,15]. Furthermore, in high-latitude and semi-arid agricultural ecosystems, thermal factors are frequently intertwined with solar radiation (Sunshine duration, SSD) [16]. High radiation drives photosynthesis and biomass accumulation, potentially exacerbating the carbon–nitrogen imbalance [17,18], and its independent contribution is rarely disentangled from thermal effects in field studies.

The inherent multicollinearity among these meteorological drivers—thermal intensity, thermal accumulation, and solar radiation—precludes accurate quantification by traditional linear regression analyses [3]. Consequently, advanced multivariate statistical frameworks, such as partial least squares regression (PLSR) and structural equation modeling (SEM), are necessitated to disentangle the complex, non-linear interdependencies governing the climate–quality relationship.

Against this background, this study conducted multi-genotype field trials across four representative spring wheat production regions in Inner Mongolia, characterized by pronounced gradients in both heat and light resources. By integrating the Zadoks phenological scale with PLSR and SEM, it aimed to: (1) quantify the relative contributions of genotypes and environments to PC variation; (2) identify the critical developmental windows where climate exerts the strongest influence; and most importantly, (3) disentangle the opposing or synergistic effects of “Thermal Intensity” versus “Thermal Accumulation” on protein synthesis. The findings provide a robust theoretical framework for optimizing wheat quality ecology by matching specific thermal regimes with crop phenology.

## 2. Results

### 2.1. Variation in Protein Content Across Environments and Genotypes

**Figure 1:**
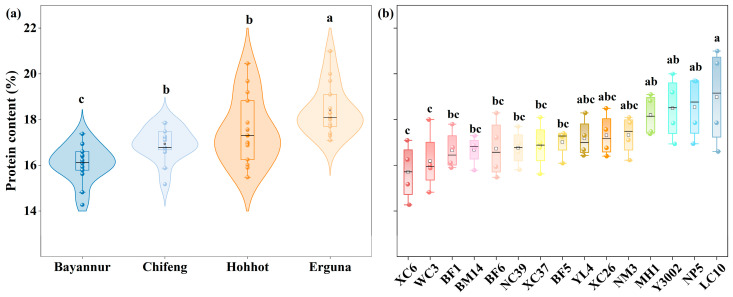
Distribution of grain protein content (%) in spring wheat affected by region and variety. (**a**) Violin plots representing the variation in protein content across four regions: Erguna, Hohhot, Chifeng, and Bayannur. (**b**) Box plots illustrating the variation in protein content among fifteen spring wheat varieties. The horizontal line within the box represents the median, the boundaries of the box indicate the 25th and 75th percentiles, and the whiskers indicate the 1.5 interquartile range. Different lowercase letters above the plots indicate significant differences at the 0.05 probability level according to Duncan’s multiple range test.

Figure 1 illustrates the distribution of PC across four distinct ecological regions and fifteen spring wheat cultivars. As depicted in the violin plots (Figure 1a), PC exhibited significant spatial heterogeneity among the experimental sites (*p* < 0.05). Specifically, the Erguna region recorded the highest mean PC at 18.53%, significantly higher than other regions. Hohhot and Chifeng followed with mean values of 17.74% and 16.81%, respectively; while both were significantly higher than Bayannur, the difference between these two intermediate regions was not statistically significant. Conversely, Bayannur exhibited the lowest PC (16.13%). Quantitatively, the mean PC in Erguna was 2.40 percentage points higher than in Bayannur, 1.72 percentage points higher than in Chifeng, and 0.79 percentage points higher than in Hohhot. The data distribution range for Erguna spans from 17.10% to 21.00%, indicating an overall higher level. In contrast, the distribution range for Bayannur ranges from 14.28% to 17.38%, reflecting a lower overall level.

Genotypic variation is presented in the box plots (Figure 1b), revealing distinct differences in protein accumulation capabilities among the cultivars. LC10 demonstrated the highest potential, achieving a mean PC of 18.98% across the four regions, followed by NP5 (18.55%) and Y3002 (18.50%). In contrast, XC6 recorded the lowest mean PC at 15.71%, followed by WC3 (16.18%). The high-protein cultivar LC10 exceeded the low-protein cultivar XC6 by a margin of 3.27 percentage points.

Two-way ANOVA further confirmed the statistical significance of these differences (Table 1) and quantified the contribution of each factor. Variety, area, and their interaction all exerted highly significant effects on PC (*p* < 0.01). The analysis of variance revealed that the environmental factor (Area) served as the predominant driver of PC variation, as evidenced by the highest F-value (F = 155.82). The genotypic factor (Variety) also played a significant role (F = 37.136), while the interaction effect, although significant, was relatively minor (F = 3.749) compared to the main effects.

### 2.2. Climatic Variations Across Different Experimental Sites

Table 2 presents the descriptive statistics for key daily climatic variables calculated based on the daily meteorological data throughout the entire spring wheat growing season (March–August) in the four regions. In terms of thermal conditions, the mean temperature (T_mean_) was highest in Bayannur (18.6 °C) and lowest in Erguna (11.3 °C). Similarly, the mean maximum temperature (T_max_) peaked in Bayannur (24.5 °C) and Hohhot (23.8 °C), while Erguna recorded the lowest value (17.7 °C). Conversely, Bayannur exhibited the highest minimum temperature (T_min_) at 10.9 °C, whereas Erguna experienced the lowest (5.4 °C). Regarding the diurnal temperature range (DTR), Bayannur and Hohhot showed substantial fluctuations (means of 13.6 °C and 14.4 °C, respectively), standing in sharp contrast to Erguna, which maintained the narrowest range (12.3 °C) but exhibited the highest maximum DTR extreme (23.8 °C). For thermal and light resources, the mean growing degree days (GDD) were highest in Bayannur (9.3 °C·d), and lowest in Erguna (5.6 °C·d). Similarly, the mean sunshine duration (SSD) was prolonged in Bayannur (9.5 h) and Chifeng (8.8 h), but was shortest in Hohhot (8.1 h) and Erguna (8.3 h). Notably, the coefficients of variation (CV) for these climatic factors varied significantly across regions, with Tmin and Erguna’s overall thermal indicators exhibiting the most pronounced variability (CV > 100%).

### 2.3. Meteorological Characteristics Across Phenological Stages

Table 3 presents the spatial variations in cumulative and average climatic factors calculated specifically for each phenological stage. Over the entire growth period (Z09–Z91), Chifeng exhibited the highest T_mean_ (20.8 °C) and T_max_ (26.3 °C), significantly exceeding those of other regions, whereas Erguna recorded the lowest T_max_ (25.4 °C). Bayannur accumulated the highest total GDD (927.2 °C·d) and SSD (895.7 h), values that were significantly greater than those observed in Erguna (731.7 °C·d and 437.2 h, respectively). Additionally, Hohhot was characterized by the largest average DTR, peaking at 15.1 °C.

Regarding specific developmental stages, during the vegetative phase (Z09–Z30), both T_mean_ and T_max_ in Chifeng and Erguna were significantly higher compared to Bayannur and Hohhot; notably, Chifeng achieved the highest GDD (144.2 °C·d) during the Z20–Z30 interval. Moving to the jointing to heading stage (Z30–Z50), Bayannur displayed significantly higher GDD and SSD than the other regions, while Erguna recorded the lowest values. A distinct and critical shift occurred during the heading to anthesis stage (Z50–Z60), where Erguna reached the highest levels for T_mean_ (22.0 °C), T_max_ (28.7 °C), and GDD (72.3 °C·d), significantly surpassing the other sites. During the late reproductive phases (Z60–Z70 and Z70–Z91), Bayannur exhibited the highest T_max_, GDD, and SSD. Conversely, Erguna showed significantly lower SSD compared to the other three regions, and recorded the lowest Tmean, Tmax, and GDD specifically during the grain-filling stage (Z70–Z91).

### 2.4. Correlation Between Protein Content and Climatic Factors

**Figure 2:**
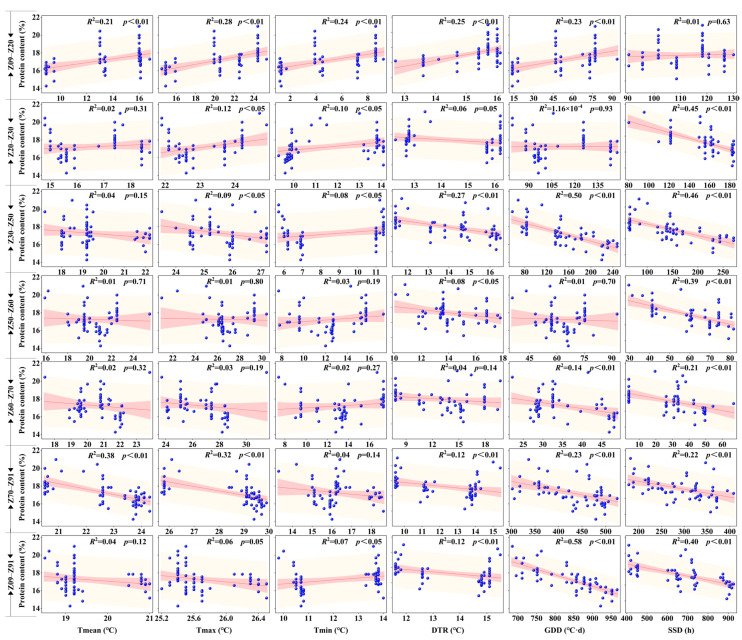
Linear regression analysis between grain protein content and climatic factors at different growth stages. Rows represent growth stages, and columns represent climatic factors. Blue dots indicate individual data points. The red solid line represents the linear regression fit, and the red shaded area indicates the 95% confidence interval. The coefficient of determination (*R*^2^) and *p*-value are displayed in each panel. Abbreviations of climatic factors are defined in Table 2. Growth stage definitions are provided in the legend of Table 3.

Figure 2 illustrates the linear regression analysis between climatic factors and PC across various growth stages. Regarding thermal indices, T_mean_ exhibited statistically significant associations with PC solely during the seedling to tillering (Z09–Z20, *R*^2^ = 0.21, *p* < 0.01) and grain filling to maturity stages (Z70–Z91, *R*^2^ = 0.38, *p* < 0.01). Similarly, T_max_ demonstrated significant associations during Z09–Z20 (*R*^2^ = 0.28, *p* < 0.01) and Z70–Z91 (*R*^2^ = 0.32, *p* < 0.01), while also reaching significance during Z20–Z50 (*p* < 0.05). In contrast, T_min_ showed significant correlations throughout the vegetative growth phase (Z09–Z50) and the whole growth period (Z09–Z91, *p* < 0.05), but no significant correlation was observed during the late reproductive phase (Z60–Z91).

DTR was significantly correlated with PC across multiple specific stages, including Z09–Z20 (*R*^2^ = 0.25, *p* < 0.01), Z30–Z50 (*R*^2^ = 0.27, *p* < 0.01), Z50–Z60 (*R*^2^ = 0.08, *p* < 0.05), and Z70–Z91 (*R*^2^ = 0.12, *p* < 0.01), as well as the whole growth period (*R*^2^ = 0.12, *p* < 0.01). For GDD, the strongest correlation was identified over the whole growth period (*R*^2^ = 0.58, *p* < 0.01). Highly significant correlations for GDD were also noted in Z30–Z50 (*R*^2^ = 0.50, *p* < 0.01), Z60–Z70 (*R*^2^ = 0.14, *p* < 0.01), and Z70–Z91 (*R*^2^ = 0.23, *p* < 0.01), whereas no significance was found in Z20–Z30 and Z50–Z60. SSD exhibited highly significant correlations (*p* < 0.01) with PC in all growth stages and the whole period except for Z09–Z20. Notably, the highest coefficient of determination for SSD was observed during the jointing to heading stage (Z30–Z50, *R*^2^ = 0.46).

### 2.5. Selection of Primary Climate Factors

**Figure 3:**
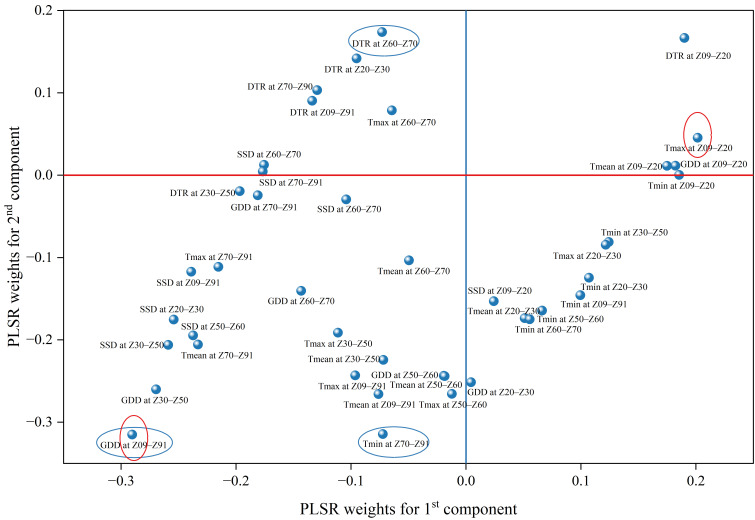
Loading plot of PLSR weights for the first and second components associated with grain protein content. Each point represents a climatic variable at a specific growth stage. The variable with the highest weight in the first component is highlighted with a red circle, and the variable with the highest weight in the second component is highlighted with a blue circle. Abbreviations of climatic factors are defined in Table 2. Growth stage definitions are provided in the legend of Table 3.

Weight analysis based on the PLSR model elucidated the relative contributions of various meteorological factors to the variation in grain protein content (Figure 3). The loading weights for the first two principal components (PC1 and PC2) indicated that the accumulated growing degree days over the whole growth period (GDD at Z09–Z91) possessed the highest loading on PC1 (highlighted by the red circle), identifying it as the predominant explanatory variable within this dimension. In contrast, the diurnal temperature ranges from seedling to tillering stages (DTR at Z09–Z20) exhibited the highest weight on PC2 (highlighted by the blue circle). Additionally, mean and maximum temperatures during the grain filling to maturity stages (T_mean_ at Z70–Z91 and T_max_ at Z70–Z91) also contributed substantially to the component space.

**Figure 4:**
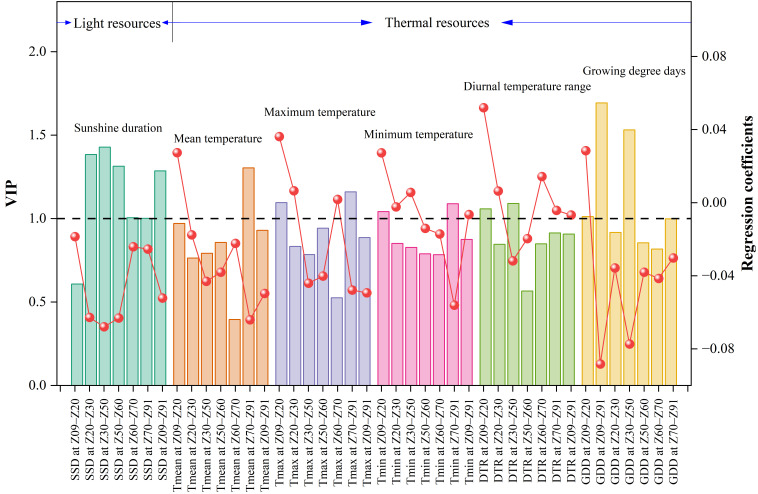
Variable importance in projection (VIP) scores and regression coefficients (RC) of climatic factors derived from the PLSR model for grain protein content. Bars represent VIP scores (left *y*-axis), and red dots represent regression coefficients (right *y*-axis). The horizontal dashed line indicates the threshold of VIP = 1. Abbreviations of climatic factors are defined in Table 2. Growth stage definitions are provided in the legend of Table 3.

The importance and directional influence of each meteorological factor were further quantified using variable importance in projection (VIP) scores and regression coefficients (RC) (Figure 4). The analysis identified 16 meteorological factors with VIP scores exceeding 1.0 as critical determinants of spring wheat protein content. Among these, GDD at Z09–Z91 recorded the highest VIP value (1.69), followed by GDD at the jointing to heading stage (Z30–Z50, VIP = 1.53) and sunshine duration (SSD at Z30–Z50, VIP = 1.43). Regarding the regression coefficients, most factors with high VIP scores exerted a negative influence. Specifically, GDD at Z09–Z91 (RC = −0.088), GDD at Z30–Z50 (RC = −0.077), and SSD at Z30–Z50 (RC = −0.068) exhibited substantial negative coefficients. Conversely, DTR and Tmax during the seedling to tillering stage (Z09–Z20) demonstrated positive effects, with RCs of 0.052 and 0.036, respectively.

### 2.6. Structural Equation Modeling and Contribution Analysis of Meteorological Factors

Based on the above analysis, 16 significant indicators with VIP > 1 were preliminarily screened. To further enhance the model’s robustness and predictive accuracy, a two-step variable selection process was implemented. First, indicators significantly correlated with grain protein content (*p* < 0.05) based on linear regression analysis (Figure 2) and possessing a VIP > 1 from the PLSR model were identified to ensure both statistical significance and explanatory power. Second, a stepwise elimination approach was applied to retain the top 10 core factors, thereby minimizing multicollinearity and maximizing the model’s predictive power (*R*^2^ = 0.637). These 10 factors including GDD at Z09–Z91, GDD at Z30–Z50, SSD at Z30–Z50, SSD at Z20–Z30, SSD at Z50–Z60, T_mean_ at Z70–Z91, SSD at Z09–Z91, T_max_ at Z70–Z91, T_max_ at Z09–Z20, and DTR at Z30–Z50, were ultimately retained for structural equation analysis.

**Figure 5:**
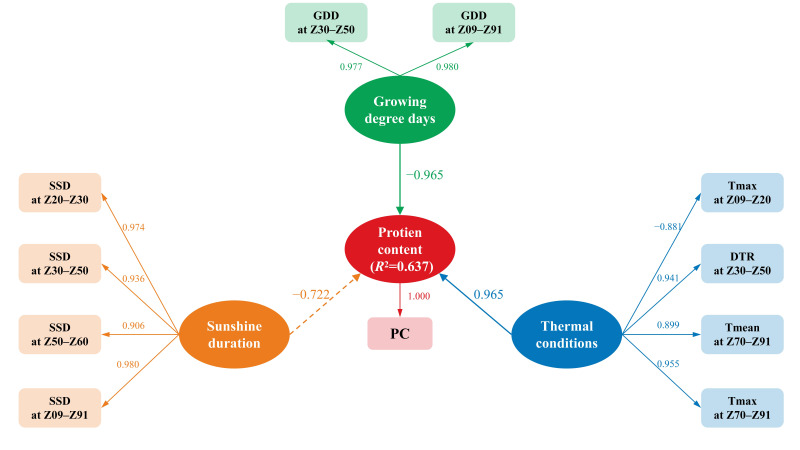
Structural equation model illustrating the direct effects of thermal conditions, growing degree days, and sunshine duration on grain protein content in spring wheat. Solid lines indicate significant paths, and dashed lines indicate non-significant paths. The coefficient of determination (*R*^2^) for protein content is shown in the central node. Abbreviations of climatic factors are defined in Table 2. Growth stage definitions are provided in the legend of Table 3.

Structural equation analysis of these key meteorological indicators revealed significant differences in the direct effects of various factors on PC (Figure 5). Specifically, “Thermal conditions” exerted a significant positive direct effect on PC, with a path coefficient of 0.965 (*p* = 0.005), while “Growing degree days” demonstrated a highly significant negative direct effect of equal magnitude (–0.965, *p* = 0.000). Although “Sunshine duration” also showed a negative association (–0.722), this effect did not reach statistical significance (*p* = 0.062). In the visual representation of the model, solid lines indicate statistically significant direct effects (*p* < 0.05), while dashed lines indicate non-significant effects (*p* ≥ 0.05). Overall, the model accounted for a substantial proportion of the variance in PC, with an *R*^2^ of 0.637.

## 3. Discussion

### 3.1. Environmental Dominance over Genotype in Determining Protein Content

This study systematically analyzed the protein content of spring wheat across four distinct ecological regions, revealing the dominant influence of environmental factors over genetic factors. While significant genetic variation was observed among cultivars (Figure 1b), the two-way ANOVA revealed that the magnitude of the environmental main effect (*F* = 155.82) was approximately fourfold greater than that of the genotypic effect (Table 1). This finding corroborates extensive literature suggesting that across broad geographical scales, environmental constraints and genotype × environment (G × E) interactions frequently overshadow singular genetic contributions [19]. Specifically, meteorological conditions during the grain-filling period have been identified as the primary drivers of variation in wheat quality traits [20], a trend observed in other cereal crops, where environmental factors account for 29–37% of the total variation in protein content [21,22,23]. It is important to note that while rainfall and nitrogen availability are known to influence grain protein content, our experimental design minimized these confounding factors. Supplemental irrigation was provided to offset natural precipitation deficits, and a uniform, non-limiting nitrogen fertilization rate (180 kg N/ha) was applied across all sites. Consequently, the observed spatial heterogeneity in protein content is primarily attributable to the distinct thermal and light regimes rather than variations in water or nitrogen supply.

Furthermore, as shown in Figure 1a, Erguna exhibits the highest protein content, while Bayannur has the lowest. This significant spatial heterogeneity is primarily attributed to the substantial differences in climatic resources between the two regions (Table 2). Conversely, the relatively lower accumulated temperature and specific phenological patterns in Erguna likely facilitated a more favorable window for nitrogen accumulation and remobilization [24]. In contrast, the high temperature and high radiation environment in Bayannur, while conducive to rapid biomass and starch deposition, likely induced a “dilution effect,” thereby compromising the final protein concentration [3,25].

Having established the predominant role of environment, we next sought to dissect how specific environmental components exert their influence. Crucially, our data reveal that not all heat is equal; the distinction between thermal intensity and thermal accumulation is paramount.

### 3.2. Thermal Intensity at Critical Windows Drives Nitrogen Remobilization via Enzymatic Regulation

It is well-established that climatic factors modulate grain chemical composition by regulating fundamental physiological processes, including photosynthesis, respiration, and nitrogen remobilization [26,27]. However, this experiment revealed that PC is driven not merely by the simple accumulation of heat units, but rather by the thermal intensity during specific critical developmental windows. The distinct temperature regimes across the four locations, particularly the thermal intensity during the Z50–Z60 stage, were identified as the primary climatic drivers of the observed spatial heterogeneity in grain protein content.

Physiologically, the Erguna recorded its highest maximum temperature (T_max_, 28.7 °C) during the heading to anthesis stage (Z50–Z60), which coincides with the critical window for nitrogen translocation from vegetative organs to grains. During this phase, the degradation of stored proteins in the flag leaf and stem, along with the subsequent re-assimilation of amino acids, is heavily dependent on the catalytic activities of glutamine synthetase (GS) and glutamate synthase (GOGAT). Previous studies indicate that the optimal catalytic temperature for GS typically ranges between 25 and 30 °C [28]. While temperatures across all four experimental sites fell within this general optimal range during the reproductive phase, the distinct advantage of the Erguna lies in the precise coupling of thermal intensity with specific phenological stages. Studies have indicated that temperature modulates wheat quality by altering the balance between starch and protein components [29]. Specifically, while daytime warming often increases protein content by suppressing starch accumulation, extreme temperatures exceeding 30 °C can impair protein quality parameters. Our study complements these findings by demonstrating that it is not merely the absolute temperature, but the precise spatiotemporal coupling of thermal intensity during the heading to anthesis stage that optimizes nitrogen remobilization, thereby maximizing protein content while avoiding the ‘dilution effect’ associated with excessive thermal accumulation.

Specifically, at the onset of nitrogen remobilization (Z50–Z60), Erguna recorded the highest maximum temperature (T_max_, 28.7 °C), which was significantly higher than Bayannur (26.6 °C). From a biochemical perspective, as GS acts as the rate-limiting enzyme in nitrogen metabolism, its catalytic velocity increases significantly with rising temperature intensity within the optimal range [30]. During this critical transition period, the thermal intensity in Erguna, which approached the peak of enzymatic reaction potential during this transitional period, likely maximized the instantaneous rates of protein degradation and amino acid mobilization in source organs. This process may also involve the induction of heat shock proteins (HSPs), thereby ensuring a more abundant nitrogen supply for the developing grain [31,32]. This physiological inference is quantitatively supported by the Structural Equation Modeling (SEM) analysis, where “Thermal Conditions” exerted a substantial positive direct effect (0.965) on PC (Figure 5). This confirms that a high-intensity thermal environment within the optimal range during early reproductive growth serves as a critical positive signal for activating nitrogen metabolic pathways.

### 3.3. Decoupling Heat Intensity from Accumulation: The Mechanism of “Dilution Effect”

However, the regulatory role of temperature on protein synthesis is not unidimensional. While high thermal intensity acts as a “catalyst” for N remobilization during anthesis, thermal accumulation (GDD) during the grain filling stage functions as a “driver” for carbon deposition. If heat appears in the form of prolonged accumulation, its role shifts from “activation” to “dilution.”

To disentangle the collinearity among complex climatic factors and quantify their causal effects, this study integrated PLSR and SEM. Consistent with the hypothesis that “excessive thermal resources lead to quality dilution,” the PLSR weight plot (Figure 3) and VIP scores (Figure 4) consistently identified whole growth period GDD (Z09–Z91) and jointing to heading GDD (Z30–Z50) as the predominant negative drivers. Notably, the Z30–Z50 stage was pinpointed as a critical window; this period not only determines sink capacity (grain number) but also represents the peak of nitrogen uptake for the wheat plant [33]. High GDD and SSD during this phase (indicated by high VIP values in Figure 4) may accelerate phenological development, thereby shortening the temporal window for N uptake and limiting the N source available for subsequent grain filling [11].

A distinct divergence was observed in the SEM analysis: the direct path coefficient of SSD on PC was not significant, whereas GDD maintained a highly significant negative effect (−0.965). This statistical discrepancy points to a deep physiological mechanism governing quality formation in semi-arid regions. In high-radiation areas like Inner Mongolia, solar radiation is typically saturating and is not the bottleneck for photosynthate accumulation. Conversely, thermal accumulation (GDD) acts as the “proximal driver” regulating the grain-filling rate.

These phenomena can be deeply explained by the theories of Carbon–Nitrogen (C-N) balance and Allometric Growth. Specifically, the extremely high GDD (486.3 °C·d) during late grain filling to maturity (Z70–Z91) in Bayannur significantly upregulated the activity of ADP-glucose pyrophosphorylase (AGPase)—the rate-limiting enzyme for starch synthesis—via thermodynamic mechanisms [34,35]. Under such conditions, the kinetic rate of starch accumulation far exceeded that of nitrogen deposition. Since starch constitutes the majority of grain dry weight, its rapid biomass expansion exerted a physical “Dilution Effect” on protein, resulting in a significant decrease in final PC [3,36]. The strong negative direct effect of GDD (−0.965) in the SEM (Figure 5) further corroborates that while SSD provides the energetic basis for photosynthesis, the allometric relationship between C and N is ultimately dictated by heat-driven enzymatic reaction rates. By maintaining high thermal intensity early on (to trigger N flow) but lower accumulated temperature during the late stages (to limit C flow), Erguna achieved a physiological balance that favored protein concentration [37].

### 3.4. Early-Stage “Priming Effect” of Diurnal Temperature Range on Root Establishment and Nitrogen Uptake

Beyond the well-established regulation of post-anthesis source–sink dynamics, our PLSR model identified the DTR during the seedling to tillering stage (Z09–Z20) as the second most significant factor of PC variations. This finding points towards an “epigenetic priming” mechanism, suggesting that early-season environmental cues can imprint lasting effects on crop physiology. A substantial DTR during early vegetative growth is hypothesized to modulate the equilibrium of endogenous phytohormones, particularly the ratio of abscisic acid (ABA) to cytokinins (CTK), thereby facilitating the establishment of a robust root system capable of penetrating deeper soil layers [38,39]. Such an enhanced root architecture serves a dual function: it not only augments the capacity for mineral nitrogen uptake from the subsoil during later reproductive stages but also regulates post-anthesis leaf senescence via root-sourced signaling, consequently elevating the nitrogen harvest index and allocation to the grain [40]. This mechanism is further corroborated by the correlation analysis (Figure 2), which revealed a significant positive correlation between Tmin during the vegetative phase and final PC. Collectively, these results demonstrate that the determination of high-protein traits in spring wheat is a synergistic process spanning the entire growth cycle; it is not solely contingent upon carbon–nitrogen competition within the “sink” during grain filling but is fundamentally initiated by the environmental adaptation and establishment of the “source” during the seedling stage [41,42].

### 3.5. Integrated Mechanistic Model of Climate-Protein Content Interactions

**Figure 6:**
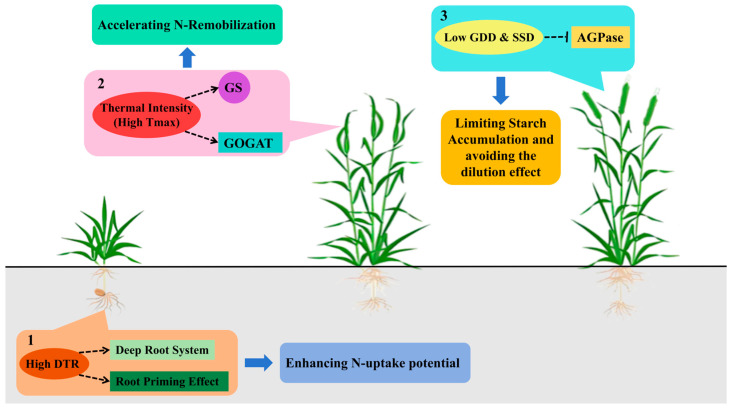
Integrating the present results with established literature, a schematic diagram was constructed to elucidate the potential mechanisms through which key climatic factors drive increases in spring wheat grain protein content: (1) Seedling to tillering (Z09–Z20): High DTR stimulates root deepening via a “priming mechanism,” augmenting N acquisition efficiency during later reproductive phases. (2) Heading to anthesis (Z50–Z60): High Tmax amplifies glutamine synthetase (GS) and glutamate synthase (GOGAT) catalytic rates, expediting vegetative N translocation to the grain sink to fuel protein accumulation. (3) Grain filling to maturity (Z70–Z91): Low GDD and SSD limit the activity of ADP-glucose pyrophosphorylase (AGPase), preventing carbohydrate over-accumulation and alleviating the “dilution effect” to maximize protein concentration. Dashed arrows indicate promoting or positive regulatory effects, whereas blunt-ended lines.

Based on the physiological and statistical evidence discussed above, a conceptual diagram illustrating the potential mechanisms by which key climatic factors enhance grain protein content in spring wheat is proposed (Figure 6): (1) Seedling to tillering (Z09–Z20): Pronounced DTR facilitates deep root system architecture through a “root priming effect,” thereby enhancing the N uptake potential in subsequent growth stages. (2) Heading to anthesis (Z50–Z60): Elevated Tmax upregulates the activities of GS and GOGAT, accelerating N remobilization from vegetative tissues to the grain and ensuring an ample N supply for protein synthesis. (3) Grain filling to maturity (Z70–Z91): Restricted GDD and SSD suppress the activity of AGPase, the rate-limiting enzyme for starch synthesis. This restricts excessive starch deposition and mitigates the “dilution effect,” consequently elevating the final grain protein concentration.

## 4. Materials and Methods

### 4.1. Site Description

**Figure 7:**
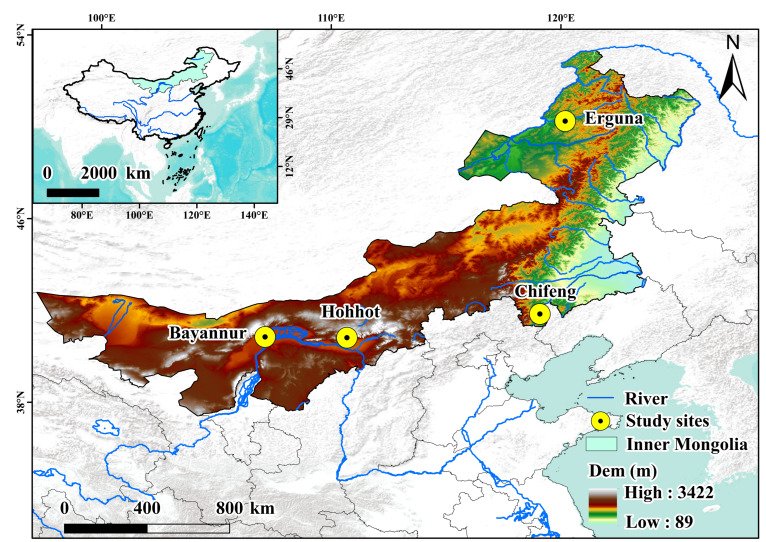
Locations and topographic maps of experimental sites. The four study sites (Bayannur, Chifeng, Hohhot, and Erguna) are indicated by yellow circles. The background map shows the digital elevation model of Inner Mongolia, with elevations ranging from 89 m to 3422 m. Major rivers are shown in blue lines. The inset map in the upper left corner shows the location of Inner Mongolia within China.

Field experiments were conducted during the 2018 cropping season at four distinct sites across the Inner Mongolia Autonomous Region, China, representing the primary spring wheat production zones. The experimental locations—Bayannur (40°45′ N, 107°25′ E), Chifeng (42°17′ N, 118°58′ E), Hohhot (40°48′ N, 111°41′ E), and Erguna (50°14′ N, 120°11′ E)—were selected to encompass a broad geographical and climatic gradient. These sites span diverse ecological zones, transitioning from temperate semi-arid climates to cold-temperate humid regimes. Specifically, Bayannur is situated within the Hetao Irrigation District; Chifeng and Hohhot represent the typical semi-arid agro-pastoral ecotone, whereas Erguna characterizes the high-latitude cold region. The geographical coordinates and spatial distribution of these study sites are visualized in Figure 7.

### 4.2. Experimental Method

The experiment selected 15 representative spring wheat varieties as test materials. These varieties exhibit diverse genetic backgrounds, with their names, origins, and parental information detailed in Table 4.

The field experiments were arranged in a randomized complete block design with three biological replicates. Each experimental plot covered an area of 2 m^2^, consisting of rows spaced 20 cm apart with a plant-to-plant spacing of 5 cm. Seeds were precision-sown at a density of 20 seeds per row. To minimize edge effects, a 1 m buffer zone of guard rows was established around the perimeter of the experimental field. Routine field management followed standard local agronomic practices for high-yield wheat production. Specifically, to decouple the confounding effects of water deficit on grain protein synthesis, supplemental irrigation was applied during critical growth stages to offset natural precipitation deficits. This ensured non-limiting water conditions throughout the reproductive period, thereby allowing climatic factors such as temperature and light to become the primary variables influencing PC. To characterize the baseline soil fertility and minimize the interference of soil heterogeneity, the physicochemical properties of the soil (0–20 cm) were measured at each site (Table A1). Furthermore, a uniform, non-limiting nitrogen fertilization rate of 180 kg N ha^−1^ was applied across all experimental sites, following standardized protocols for multi-environment wheat quality trials to ensure that nitrogen availability was not a limiting factor for grain protein content [3].

### 4.3. Data Collection and Measurement

#### 4.3.1. Meteorological Data Acquisition

**Figure 8:**
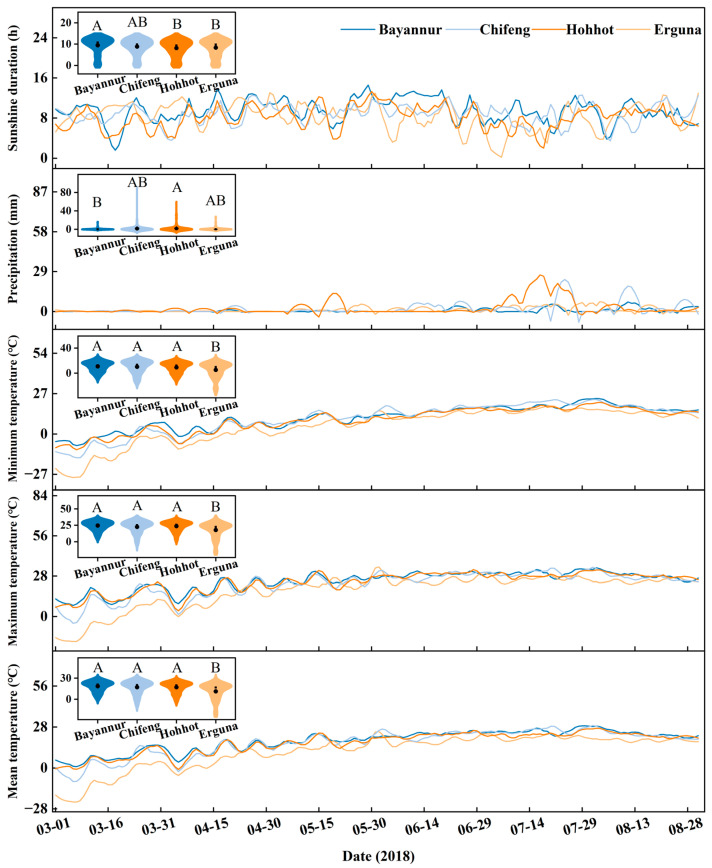
Dynamics of meteorological factors during the spring wheat growing season (March–August) in Bayannur, Chifeng, Hohhot, and Erguna. The insets illustrate the overall climatic variations across the entire growth period. Different letters in the insets indicate significant differences among regions (*p *< 0.05).

Daily meteorological datasets covering the entire 2018 spring wheat growing season (spanning March through August) were acquired from the China Meteorological Administration (CMA). The dataset comprised five key climatic variables: maximum temperature (T_max_), minimum temperature (T_min_), mean temperature (T_mean_), precipitation, and sunshine duration (SSD). The temporal dynamics and spatial heterogeneity of these environmental factors across the four experimental sites are illustrated in Figure 8.

#### 4.3.2. Determination of Protein Content

At physiological maturity, wheat spikes were manually harvested and air-dried to a constant weight under ambient conditions. Following this, mechanical threshing was performed to obtain clean grain samples. Prior to chemical analysis, the grains were milled into a fine powder using a laboratory cyclone mill and passed through a 0.5 mm sieve. Total grain nitrogen content was then determined using the standard micro-Kjeldahl method. Subsequently, protein content was calculated by multiplying the nitrogen concentration by a conversion factor of 5.7. To ensure analytical precision, all measurements were performed in two technical replicates.

### 4.4. Calculation Methods and Statistical Analysis

#### 4.4.1. Calculation of Meteorological Indices

Based on raw meteorological data, calculate key meteorological indicators. Specifically, the Diurnal Temperature Range (DTR) was calculated using the following equation [43]:

(1) DTR=Tmax−Tmin,

Heat accumulation throughout the wheat growth period was characterized using growing degree days (GDD), also referred to as Effective Accumulated Temperature (EAT). The GDD values were calculated according to the method described by using the following equation [44]:

(2) $$GDD=\sum_{i=d1}^{dn}(Tmean,i-Tbase)$$

where d_1_ and dn represent the first and last day of the specific growth stage, respectively; T_mean,i_ denotes the daily mean temperature for day i; and T_base_ is the base temperature for spring wheat growth. T_base_ was set at 10 °C. To ensure biological relevance, if T_mean,i_ < T_base_, the daily GDD value was recorded as zero.

#### 4.4.2. Statistical Analysis

Raw data were organized and preprocessed using Microsoft Excel. A two-way analysis of variance (ANOVA) was performed using IBM SPSS Statistics 26 to evaluate the main effects of variety, area, and their interaction on PC. Pearson correlation analysis was also conducted using SPSS to identify relationships between key climate factors and quality traits. Multivariate analysis and data visualization were executed using SIMCA 14.1 and Origin 2025b. Furthermore, partial least squares structural equation modeling (PLS-SEM) was constructed using SmartPLS 4.1 to quantify the causal pathways driving PC variation. Schematic diagrams of the structural models and conceptual diagrams were refined using Microsoft PowerPoint 2021.

## 5. Conclusions

This study establishes that in semi-arid regions, environmental factors are the primary drivers of spring wheat protein content variation, exerting a significantly greater influence than genotype. The core contribution of this study lies in proposing a “Thermal Intensity versus Accumulation” theoretical framework for quality ecology: specifically, higher thermal intensity during heading to anthesis (Z50–Z60) acts as a positive signal to activate nitrogen remobilization via optimized enzymatic kinetics, whereas excessive accumulated heat (GDD) and radiation during grain filling to maturity (Z70–Z91) drive a “dilution effect” by disproportionately accelerating starch deposition. Additionally, the diurnal temperature range at the seedling stage was identified as a critical early predictor, suggesting a physiological “priming” effect on root establishment. In conclusion, high-quality wheat production necessitates a precise spatiotemporal coupling of climatic resources—synergizing “high thermal intensity for nitrogen mobilization” with “moderate thermal accumulation to mitigate carbohydrate dilution.” Future breeding and management strategies must prioritize aligning crop phenology with these optimal thermal windows to reconcile the trade-off between yield and quality under a warming climate.

## Figures and Tables

**Table 1:** Two-way ANOVA for the effects of variety, area, and their interaction on grain protein content.

Indicator	Variety		Area		Variety × Area	
	F-Value	*p*-Value	F-Value	*p*-Value	F-Value	*p*-Value
Protein content (%)	37.136	*p* < 0.01	155.82	*p* < 0.01	3.749	*p* < 0.01

The significant interaction effect (Variety × Area, *p* < 0.01) indicates that the protein accumulation potential of the tested cultivars was significantly modulated by the specific climatic conditions of each experimental site.

**Table 2:** Descriptive statistics of major climatic factors during the whole growth period of spring wheat (March–August) in four regions.

Area	Indicator	Abridge	Maximum Value	Minimum Value	Mean Value	Standard Deviation	Range	Coefficient of Variation (%)
Bayannur	Mean temperature (°C)	T_mean_	30.4	−2.0	18.6	7.5	32.4	40.3
	Maximum temperature (°C)	T_max_	36.2	3.5	24.5	7.4	32.7	30.0
	Minimum temperature (°C)	T_min_	25.0	−9.9	10.9	8.0	34.9	73.5
	Diurnal temperature range (°C)	DTR	23.8	3.9	13.6	4.4	19.9	32.3
	Growing degree days (°C·d)	GDD	20.4	0.0	9.3	6.1	20.4	65.4
	Sunshine duration (h)	SSD	14.2	0.0	9.5	4.0	14.2	42.3
Chifeng	Mean temperature (°C)	T_mean_	30.0	−12.2	17.2	9.4	42.2	54.8
	Maximum temperature (°C)	T_max_	35.5	−9.1	22.6	9.5	44.6	41.9
	Minimum temperature (°C)	T_min_	23.3	−18.3	10.3	10.3	41.6	100.8
	Diurnal temperature range (°C)	DTR	22.6	1.8	12.4	4.4	20.8	35.3
	Growing degree days (°C·d)	GDD	20.0	0.0	8.9	6.3	20.0	70.3
	Sunshine duration (h)	SSD	14.3	0.0	8.8	3.8	14.3	43.2
Hohhot	Mean temperature (°C)	T_mean_	27.6	−4.2	17.2	7.8	31.8	45.2
	Maximum temperature (°C)	T_max_	34.0	0.0	23.8	7.6	34.0	32.2
	Minimum temperature (°C)	T_min_	21.7	−13.0	9.4	8.4	34.7	89.2
	Diurnal temperature range (°C)	DTR	24.0	4.3	14.4	3.7	19.7	25.9
	Growing degree days (°C·d)	GDD	17.6	0.0	8.3	5.7	17.6	68.0
	Sunshine duration (h)	SSD	14.1	0.0	8.1	3.9	14.1	48.3
Erguna	Mean temperature (°C)	T_mean_	27.2	−24.4	11.3	12.0	51.6	106.4
	Maximum temperature (°C)	T_max_	36.2	−18.2	17.7	12.2	54.4	69.1
	Minimum temperature (°C)	T_min_	20.9	−31.7	5.4	12.2	52.6	227.9
	Diurnal temperature range (°C)	DTR	23.8	4.0	12.3	3.7	19.8	29.8
	Growing degree days (°C·d)	GDD	17.2	0.0	5.6	5.0	17.2	88.4
	Sunshine duration (h)	SSD	13.9	0.0	8.3	4.1	13.9	48.5

**Table 3:** Climate conditions during different stages of growth across four regions.

Duration	Area	T_mean_^b^ (°C)	T_max_ (°C)	T_min_ (°C)	DTR (°C)	GDD (°C·d)	SSD (h)
Z09–Z20 ^a^	Bayannur	9.1 ± 0.28 c ^c^	15.2 ± 0.48 d	1.3 ± 0.13 d	13.9 ± 0.59 d	14.8 ± 1.92 d	111.6 ± 5.69 c
	Chifeng	16.1 ± 0.00 a	22.5 ± 0.08 b	7.2 ± 0.04 b	15.4 ± 0.00 c	71.8 ± 2.28 b	126.6 ± 0.00 a
	Hohhot	13.2 ± 0.19 b	20.2 ± 0.31 c	4.2 ± 0.29 c	16.0 ± 0.04 a	48.1 ± 1.95 c	98.1 ± 4.21 d
	Erguna	16.1 ± 0.21 a	24.5 ± 0.23 a	8.6 ± 0.15 a	15.7 ± 0.10 b	78.1 ± 4.34 a	119.1 ± 3.05 b
Z20–Z30	Bayannur	15.7 ± 0.15 c	22.6 ± 0.10 c	6.9 ± 0.07 c	15.8 ± 0.09 d	97.2 ± 2.35 c	182.7 ± 0.63 a
	Chifeng	18.5 ± 0.12 a	23.8 ± 0.12 b	11.0 ± 0.00 b	12.8 ± 0.14 c	144.2 ± 1.78 a	154.3 ± 2.44 c
	Hohhot	15.1 ± 0.23 d	22.2 ± 0.18 d	6.0 ± 0.18 d	16.2 ± 0.00 a	90.1 ± 5.68 d	161.5 ± 5.01 d
	Erguna	17.4 ± 0.19 b	24.2 ± 0.31 a	11.4 ± 0.07 a	12.9 ± 0.29 c	121.0 ± 10.15 b	116.1 ± 12.27 b
Z30–Z50	Bayannur	19.2 ± 0.11 b	25.9 ± 0.06 a	9.8 ± 0.20 b	16.2 ± 0.18 a	219.0 ± 25.15 a	230.1 ± 31.50 a
	Chifeng	21.5 ± 1.21 a	26.7 ± 1.11 a	13.4 ± 1.15 a	13.3 ± 0.31 c	160.7 ± 38.26 b	137.4 ± 25.19 b
	Hohhot	18.3 ± 0.47 c	24.8 ± 0.66 b	10.0 ± 0.42 b	14.8 ± 0.33 b	136.8 ± 18.37 b	137.4 ± 23.79 b
	Erguna	19.0 ± 0.55 b	25.1 ± 0.47 b	13.6 ± 0.62 a	11.5 ± 0.17 d	79.3 ± 7.63 c	67.1 ± 5.35 c
Z50–Z60	Bayannur	20.5 ± 0.96 b	26.6 ± 0.55 b	11.7 ± 1.51 c	14.9 ± 1.26 b	63.3 ± 5.79 b	70.3 ± 8.47 a
	Chifeng	20.7 ± 2.18 abc	26.7 ± 1.68 b	13.9 ± 1.71 b	12.8 ± 0.66 c	64.2 ± 13.06 b	60.2 ± 1.07 b
	Hohhot	19.0 ± 1.42 c	25.7 ± 1.89 b	9.6 ± 0.82 d	16.2 ± 1.56 a	55.1 ± 9.54 c	70.0 ± 10.91 a
	Erguna	22.0 ± 1.02 a	28.7 ± 1.44 a	15.5 ± 0.39 a	13.2 ± 1.11 c	72.3 ± 6.18 a	40.0 ± 3.97 c
Z60–Z70	Bayannur	21.5 ± 0.87 a	27.8 ± 1.03 a	13.0 ± 0.87 c	14.8 ± 0.66 b	46.5 ± 2.47 a	49.1 ± 5.89 a
	Chifeng	20.2 ± 1.23 bc	25.5 ± 1.75 b	13.9 ± 0.86 b	11.6 ± 1.14 c	30.5 ± 3.71 bc	28.4 ± 2.44 c
	Hohhot	19.6 ± 0.70 c	27.0 ± 1.12 a	9.6 ± 1.03 d	17.5 ± 1.41 a	28.8 ± 2.12 c	33.5 ± 28.5 b
	Erguna	21.1 ± 0.77 ab	25.8 ± 1.73 b	17.0 ± 0.85 a	8.7 ± 2.27 d	34.8 ± 4.74 b	6.7 ± 8.27 d
Z70–Z91	Bayannur	23.8 ± 0.18 a	29.6 ± 0.19 a	15.9 ± 0.20 c	13.7 ± 0.12 b	486.3 ± 21.11 a	363.5 ± 21.14 a
	Chifeng	23.8 ± 0.44 a	29.2 ± 0.36 b	18.0 ± 0.54 a	11.2 ± 0.24 c	401.8 ± 35.36 b	262.8 ± 22.39 c
	Hohhot	22.5 ± 0.20 b	29.1 ± 0.14 b	14.9 ± 0.53 d	14.2 ± 0.44 a	476.3 ± 20.09 a	321.9 ± 19.78 b
	Erguna	20.6 ± 0.18 c	25.9 ± 0.18 c	16.3 ± 0.11 b	9.5 ± 0.08 d	346.2 ± 22.22 c	207.2 ± 18.52 d
Z09–Z91	Bayannur	19.2 ± 0.14 b	25.5 ± 0.15 bc	10.7 ± 0.11 b	14.8 ± 0.07 b	927.2 ± 26.23 a	895.7 ± 18.72 a
	Chifeng	20.8 ± 0.16 a	26.3 ± 0.13 a	13.7 ± 0.21 a	12.6 ± 0.09 c	873.1 ± 41.96 b	643.1 ± 19.92 c
	Hohhot	18.9 ± 0.16 c	25.6 ± 0.14 b	10.5 ± 0.27 b	15.1 ± 0.12 a	835.1 ± 41.77 c	724.3 ± 20.99 b
	Erguna	19.2 ± 0.00 b	25.4 ± 0.04 c	13.8 ± 0.06 a	11.7 ± 0.10 d	731.7 ± 26.60 d	437.2 ± 19.12 d

^a^ Z09–Z20, seedling to tillering; Z20–Z30, tillering to jointing; Z30–Z50, jointing to heading; Z50–Z60, heading to anthesis; Z60–Z70, anthesis to grain filling; Z70–Z91, grain filling to maturity; Z09–Z91, whole growth period. ^b^ Tmean, Tmax, Tmin, DTR, GDD), and SSD represent the mean temperature, maximum temperature, minimum temperature, diurnal temperature range, growing degree days, and sunshine duration. ^c^ Values are presented as mean ± standard deviation. Different lowercase letters within the same column and growth stage indicate significant differences among regions at the 0.05 probability level according to Duncan’s multiple range test.

**Table 4:** Pedigree information and geographical origin of the 15 spring wheat cultivars used in this study.

Serial Number	Variety Name	Female/Male	Varietal Origin
1	XC6	Orofen/76-26	Urumqi, Xinjiang, China
2	WC3	Wuchun 2/8024	Wuwei, Gansu, China
3	BF1	Yongliang 4/90-13-2	Bayannur, Inner Mongolia, China
4	BM14	Bamai 13/2003-18	Bayannur, Inner Mongolia, China
5	BF6	Yongliang 4/96-205	Bayannur, Inner Mongolia, China
6	NC39	Ningchun 4/90W18	Yinchuan, Ningxia, China
7	XC37	Xinchun 22/01-26	Urumqi, Xinjiang, China
8	BF5	Yongliang 4/96-205	Bayannur, Inner Mongolia, China
9	YL4	7606/7914	Bayannur, Inner Mongolia, China
10	XC26	96-12/96-43	Urumqi, Xinjiang, China
11	NM3	81-39-2/81-18	Urumqi, Xinjiang, China
12	MH1	K420/7606 (Antherculture)	Hohhot, Inner Mongolia, China
13	Y3002	Yongliang 4/96-205	Bayannur, Inner Mongolia, China
14	NP5	Yongliang 4/90-13-2	Bayannur, Inner Mongolia, China
15	LC10	Liaochun 9/7846	Shenyang, Liaoning, China

## Data Availability

The original contributions presented in this study are included in the article. Further inquiries can be directed to the corresponding authors.

## Abbreviations

The following abbreviations are used in this manuscript:

Tmean	Mean temperature
Tmax	Maximum temperature
Tmin	Minimum temperature
DTR	Diurnal temperature range
GDD	Growing degree days
SSD	Sunshine duration
Z09–Z20	Seedling to tillering
Z20–Z30	Tillering to jointing
Z30–Z50	Jointing to heading
Z50–Z60	Heading to anthesis
Z60–Z70	Anthesis to grain filling
Z70–Z91	Grain filling to maturity
Z09–Z91	Whole growth period
VIP	Variable importance in projection
RC	Regression coefficients
PLSR	Partial least squares regression
SEM	Structural equation modeling
PC	Protein content
GS	Glutamine synthetase
GOGAT	Glutamate synthase

## Appendix A

**Table A1:** Physicochemical properties of the soil (0–20 cm) at the four experimental sites.

Area	Soil Type	Available N (mg kg^−1^)	Available P (mg kg^−1^)	Available K (mg kg^−1^)	PH
Bayannur	Loam	102.0	32.8	140.0	8.5
Chifeng	Loam	42.0	2.6	72.0	7.9
Hohhot	Sandy loam	59.5	16.0	117.5	8.2
Erguna	Chernozem	144.2	22.52	85.3	7.2

## References

[1] Wang D., Li F., Cao S., Zhang K. (2020). Genomic and functional genomics analyses of gluten proteins and prospect for simultaneous improvement of end-use and health-related traits in wheat. Theor. Appl. Genet..

[2] Lama S., Muneer F., America A.H.P., Kuktaite R. (2025). Polymeric gluten proteins as climate-resilient markers of quality: Can LC-MS/MS provide valuable information about spring wheat grown in diverse climates?. J. Agric. Food Chem..

[3] Asseng S., Martre P., Maiorano A., Rötter R.P., O’Leary G.J., Fitzgerald G.J., Girousse C., Motzo R., Giunta F., Babar M.A. (2019). Climate change impact and adaptation for wheat protein. Glob. Change Biol..

[4] Asseng S., Ewert F., Martre P., Rötter R.P., Lobell D.B., Cammarano D., Kimball B.A., Ottman M.J., Wall G.W., White J.W. (2015). Rising temperatures reduce global wheat production. Nat. Clim. Change.

[5] Laidig F., Hüsken A., Rentel D., Piepho H.-P. (2022). Protein use efficiency and stability of baking quality in winter wheat based on the relation of loaf volume and grain protein content. Theor. Appl. Genet..

[6] Gadissa F., Gudeta T.B. (2023). Phenotypic characterization and seed viability test in ex-situ conserved ethiopian cultivated barley (*Hordeum vulgare* L.) landraces. BMC Plant Biol..

[7] Zhang X., Guo Z., Xu J., Huang C., Dang H., Mu W., Zhang L., Hou S., Huang N., Li C. (2024). Nutrient requirements determined by grain yield and protein content to optimize N, P, and K fertilizer management in China. Sci. Total Environ..

[8] Liu X., Hu B., Chu C. (2022). Nitrogen assimilation in plants: Current status and future prospects. J. Genet. Genom..

[9] Fotouo Makouate H., Zude-Sasse M. (2025). Advances in growing degree days models for flowering to harvest: Optimizing crop management with methods of precision horticulture—A review. Horticulturae.

[10] Sehgal A., Sita K., Siddique K.H.M., Kumar R., Bhogireddy S., Varshney R.K., Rao B.H., Nair R.M., Prasad P.V.V., Nayyar H. (2018). Drought or/and heat-stress effects on seed filling in food crops: Impacts on functional biochemistry, seed yields, and nutritional quality. Front. Plant Sci..

[11] Kashyap S., Reddy B.H.R., Devi S., Kurpad A.V. (2024). Potential impact of climate change on dietary grain protein content and its bioavailability-a mini review. Front. Nutr..

[12] Tegeder M., Masclaux-Daubresse C. (2018). Source and sink mechanisms of nitrogen transport and use. New Phytol..

[13] Daniel R.M., Danson M.J. (2010). A new understanding of how temperature affects the catalytic activity of enzymes. Trends Biochem. Sci..

[14] Verslues P.E., Bailey-Serres J., Brodersen C., Buckley T.N., Conti L., Christmann A., Dinneny J.R., Grill E., Hayes S., Heckman R.W. (2022). Burning questions for a warming and changing world: 15 unknowns in plant abiotic stress. Plant Cell.

[15] Wang H., Jia Y., Bai X., Gong W., Liu G., Wang H., Xin J., Wu Y., Zheng H., Liu H. (2024). Whole-transcriptome profiling and functional prediction of long non-coding RNAs associated with cold tolerance in *Japonica* rice varieties. Int. J. Mol. Sci..

[16] Suehrcke H., Bowden R.S., Hollands K.G.T. (2013). Relationship between sunshine duration and solar radiation. Sol. Energy.

[17] Honda S., Ohkubo S., San N.S., Nakkasame A., Tomisawa K., Katsura K., Ookawa T., Nagano A.J., Adachi S. (2021). Maintaining higher leaf photosynthesis after heading stage could promote biomass accumulation in rice. Sci. Rep..

[18] Zhai P., Cheng R., Gong Z., Huang J., Yang X., Zhang X., Zhao X. (2025). Plant biomass allocation-regulated nitrogen and phosphorus addition effects on ecosystem carbon fluxes of a lucerne (*Medicago Sativa* ssp. *Sativa*) plantation in the Loess Plateau. Plants.

[19] Mohammadi R., Roostaei M., Armion M., Abdipour M., Rahmati M., Shahbazi K. (2025). Deciphering genotype × environment interaction for grain yield in durum wheat: An integration of analytical and empirical approaches for increased yield stability and adaptability. Eur. J. Agron..

[20] Lama S., Leiva F., Vallenback P., Chawade A., Kuktaite R. (2023). Impacts of heat, drought, and combined heat-drought stress on yield, phenotypic traits, and gluten protein traits: Capturing stability of spring wheat in excessive environments. Front. Plant Sci..

[21] Kunjaroenruk J., Koonmanee S., Singkham N., Chankaew S., Suriharn K. (2025). Genotypic variation and seasonal effects on rice (*Oryza sativa* L.) grain protein content and yield in tropical savannah environment. J. Agric. Food Res..

[22] Tanin M.J., Sharma A., Saini D.K., Singh S., Kashyap L., Srivastava P., Mavi G.S., Kaur S., Kumar V., Kumar V. (2022). Ascertaining yield and grain protein content stability in wheat genotypes having the Gpc-B1 gene using univariate, multivariate, and correlation analysis. Front. Genet..

[23] Gomez-Becerra H.F., Yazici A., Ozturk L., Budak H., Peleg Z., Morgounov A., Fahima T., Saranga Y., Cakmak I. (2010). Genetic variation and environmental stability of grain mineral nutrient concentrations in *Triticum dicoccoides* under five environments. Euphytica.

[24] Lu L., Wang Q., Zhang W., Gao M., Xv Y., Li S., Dong H., Chen D., Yan P., Dong Z. (2024). Urea coated with polyaspartic acid-chitosan increases foxtail millet (*Setaria italica* L. Beauv.) grain yield by improving nitrogen metabolism. Plants.

[25] Marcos-Barbero E.L., Pérez P., Martínez-Carrasco R., Arellano J.B., Morcuende R. (2021). Genotypic variability on grain yield and grain nutritional quality characteristics of wheat grown under elevated CO_2_ and high temperature. Plants.

[26] Luo L., Hui X., He G., Wang S., Wang Z., Siddique K.H.M. (2022). Benefits and limitations to plastic mulching and nitrogen fertilization on grain yield and sulfur nutrition: Multi-site field trials in the semiarid area of China. Front. Plant Sci..

[27] Lin Z., Zhang X., Wang Z., Jiang Y., Liu Z., Alexander D., Li G., Wang S., Ding Y. (2017). Metabolomic analysis of pathways related to rice grain chalkiness by a notched-belly mutant with high occurrence of white-belly grains. BMC Plant Biol..

[28] Kong L., Xie Y., Hu L., Feng B., Li S. (2016). Remobilization of vegetative nitrogen to developing grain in wheat (*Triticum aestivum* L.). Field Crops Res..

[29] Han P., Wang Y., Sun H. (2025). Impact of temperature stresses on wheat quality: A focus on starch and protein composition. Foods.

[30] Ru C., Hu X., Wang W., Yan H. (2024). Impact of nitrogen on photosynthesis, remobilization, yield, and efficiency in winter wheat under heat and drought stress. Agric. Water Manag..

[31] Duan M., Tao M., Wei F., Liu H., Han S., Feng J., Ran Q., Duan X., He Z., Yang S. (2025). *Leucocalocybe mongolica* fungus enhances rice growth by reshaping root metabolism, and hormone-associated pathways. Rice.

[32] Li J., Chauve L., Phelps G., Brielmann R.M., Morimoto R.I. (2016). E2F coregulates an essential HSF developmental program that is distinct from the heat-shock response. Genes Dev..

[33] Li C., Shi Y., Yu Z., Zhang Y., Zhang Z. (2025). Optimizing nitrogen application strategies can improve grain yield by increasing dry matter translocation, promoting grain filling, and improving harvest indices. Front. Plant Sci..

[34] Vishwakarma C., Krishna G.K., Kapoor R.T., Mathur K., Dalal M., Singh N.K., Mohapatra T., Chinnusamy V. (2023). Physiological analysis of source-sink relationship in rice genotypes with contrasting grain yields. Plants.

[35] Boehlein S.K., Sewell A.K., Cross J., Stewart J.D., Hannah L.C. (2005). Purification and characterization of adenosine diphosphate glucose pyro phosphorylase from maize/potato mosaics. Plant Physiol..

[36] Fradgley N.S., Bacon J., Bentley A.R., Costa-Neto G., Cottrell A., Crossa J., Cuevas J., Kerton M., Pope E., Swarbreck S.M. (2023). Prediction of near-term climate change impacts on UK wheat quality and the potential for adaptation through plant breeding. Glob. Change Biol..

[37] Fei L., Pan Y., Ma H., Guo R., Wang M., Ling N., Shen Q., Guo S. (2024). Optimal organic-inorganic fertilization increases rice yield through source-sink balance during grain filling. Field Crops Res..

[38] Calleja-Cabrera J., Boter M., Oñate-Sánchez L., Pernas M. (2020). Root growth adaptation to climate change in crops. Front. Plant Sci..

[39] Yun F., Liu H., Deng Y., Hou X., Liao W. (2023). The role of light-regulated auxin signaling in root development. Int. J. Mol. Sci..

[40] Schmidt J.E., Gaudin A.C.M. (2017). Toward an integrated root ideotype for irrigated systems. Trends Plant Sci..

[41] Mumtahina N., Matsuoka A., Uga Y., Shimono H., Matsunami M. (2026). Yield performance of rice with different root system architecture with combination of *DRO1* and *qSOR1* alleles under different fertilization regimes. Field Crops Res..

[42] Venzhik Y., Deryabin A., Popov V., Dykman L., Moshkov I. (2022). Priming with gold nanoparticles leads to changes in the photosynthetic apparatus and improves the cold tolerance of wheat. Plant Physiol. Biochem..

[43] van Etten J., de Sousa K., Aguilar A., Barrios M., Coto A., Dell’Acqua M., Fadda C., Gebrehawaryat Y., van de Gevel J., Gupta A. (2019). Crop variety management for climate adaptation supported by citizen science. Proc. Natl. Acad. Sci. USA.

[44] McMaster G.S., Wilhelm W.W. (1997). Growing degree-days: One equation, two interpretations. Agric. For. Meteorol..

